# Effect of Acupuncture on the p38 Signaling Pathway in Several Nervous System Diseases: A Systematic Review

**DOI:** 10.3390/ijms21134693

**Published:** 2020-06-30

**Authors:** Tzu-Hsuan Wei, Ching-Liang Hsieh

**Affiliations:** 1 Department of Chinese Medicine, China Medical University Hospital, Taichung 40447, Taiwan; u102030015@cmu.edu.tw; 2 Chinese Medicine Research Center, China Medical University, Taichung 40402, Taiwan; 3 Graduate Institute of Acupuncture Science, College of Chinese Medicine, China Medical University, Taichung 40402, Taiwan

**Keywords:** acupuncture, p38 signaling pathway, nervous system diseases

## Abstract

Acupuncture is clinically used to treat various diseases and exerts positive local and systemic effects in several nervous system diseases. Advanced molecular and clinical studies have continually attempted to decipher the mechanisms underlying these effects of acupuncture. While a growing understanding of the pathophysiology underlying several nervous system diseases shows it to be related to inflammation and impair cell regeneration after ischemic events, the relationship between the therapeutic mechanism of acupuncture and the p38 MAPK signal pathway has yet to be elucidated. This review discusses the latest advancements in the identification of the effect of acupuncture on the p38 signaling pathway in several nervous system diseases. We electronically searched databases including PubMed, Embase, and the Cochrane Library from their inception to April 2020, using the following keywords alone or in various combinations: “acupuncture”, “p38 MAPK pathway”, “signaling”, “stress response”, “inflammation”, “immune”, “pain”, “analgesic”, “cerebral ischemic injury”, “epilepsy”, “Alzheimer’s disease”, “Parkinson’s disease”, “dementia”, “degenerative”, and “homeostasis”. Manual acupuncture and electroacupuncture confer positive therapeutic effects by regulating proinflammatory cytokines, ion channels, scaffold proteins, and transcription factors including TRPV1/4, Na_v_, BDNF, and NADMR1; consequently, p38 regulates various phenomena including cell communication, remodeling, regeneration, and gene expression. In this review article, we found the most common acupoints for the relief of nervous system disorders including GV20, GV14, ST36, ST37, and LI4. Acupuncture exhibits dual regulatory functions of activating or inhibiting different p38 MAPK pathways, contributing to an overall improvement of clinical symptoms and function in several nervous system diseases.

## 1. Introduction 

Acupuncture is a form of therapy that has been practiced for more than 3000 years in Asia [1,2]. Medical doctors practice acupuncture under the guidance of meridian theory, which was first recorded in detail in The Yellow Emperor’s Classic of Internal Medicine [2]. To perform acupuncture, doctors use thin and sterile metal needles to penetrate specific stimulation points termed acupoints, and they manipulate the needle to achieve “de qi” status [2]. Both manual and electroacupuncture (EA) are used in medical practice. Acupuncture is generally a safe, easy to perform, [3,4,5] and economical procedure that provides another choice for those who are concerned about the adverse effects of routine managements such as drug prescription [6,7]. Occasional minor problems, such as needles left in patients by mistake, headaches, and drowsiness have been reported after acupuncture treatment; usually, these symptoms are self-resolved after a short rest [1,3,6,7]. Due to the limited number of controlled clinical trials that have been published, the efficacy of acupuncture as treatment for has been questioned [8].

**Figure 1:**
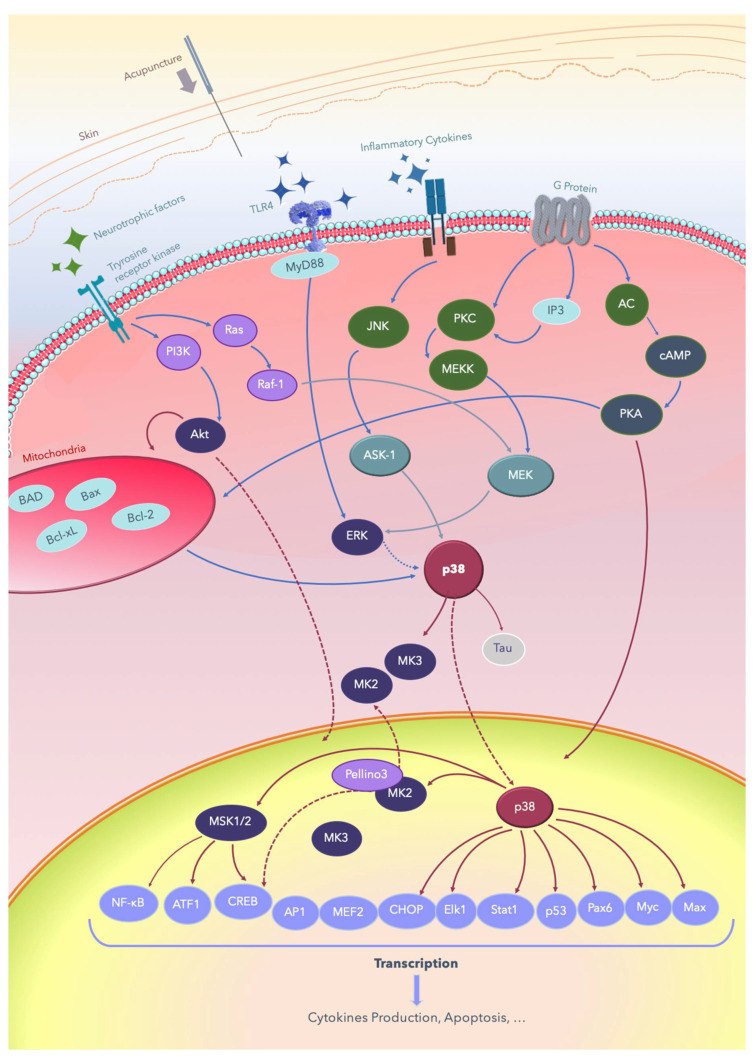
Summary of the signal transduction pathways through which acupuncture treats nervous system diseases. Acupuncture is applied on acupoints and results in de qi, evoking the excitation of cell membrane receptors, such as the Tyrosine receptor kinase and TLR/ligand, and subsequently producing signal transduction. AC: adenyl cyclase; Akt: protein kinase B; AMPK: AMP-activated protein kinase; ASK-1: apoptosis signal-regulating kinase 1; Bad: Bcl-2-associated death promoter; Bax: Bcl-2 associated X; Bcl-2: B-cell lymphoma 2; Bcl2-xl: B-cell lymphomaextralarge; cAMP: cyclic adenosine monophosphate; CREB: cAMP response element-binding protein; ERK: extracellular signal-regulated kinase; IP3: inositol triphosphate; JNK: c-Jun N-terminal kinases; Elk-1: erythroblast transformation specific (ETS) like-1 protein; Max: a transcription factor coded by the myc-associated factor X; MEF: myocyte-enhancing factor; MEK: MEK kinase; MEKK: MK kinase kinase; MSK: mitogen- and stress-activated protein kinase; Myc: a group of transcription factors coded by regulator genes and a proto-oncogene called Myc; MyD88: myeloid differentiation primary response 88; TLRs: Toll-like receptors; NF-κB: nuclear factor kappa B; Pax6: a paired-box protein encoded by the master gene Pax-6; PI3K: phosphatidylinositol-4,5-bisphosphate 3-kinase; PKA: protein kinase A; PKC: protein kinase C; ATF: activating transcription factor; AP-1: activator protein; CHOP: C/EBP homologous; Stat1: signal transducer and activator of transcription 1.

Acupuncture is widely used to treat various diseases and exerts positive effects, including analgesia, at both local and systemic levels [3,8,9,10,11,12]; it improves consciousness and cognition [13,14,15,16,17,18,19,20], and it induces therapeutic effects in several nervous system diseases [8,13,14,15,16,17,18,19,20]. Advanced molecular and clinical studies have continually attempted to decipher the mechanisms underlying these effects of acupuncture [2,7,16,17,20,21,22,23,24,25,26,27,28,29,30,31,32,33,34,35,36,37,38,39,40,41,42,43,44,45,46,47,48,49,50,51,52,53,54,55,56,57,58,59,60,61,62,63,64,65,66,67,68,69,70,71,72]. The signal transduction pathways through which acupuncture treats nervous system diseases involves multiple signal pathways, including p38 mitogen-activated protein kinases (p38 MAPKs) [7,33,34,35,36,37,38,39], Raf/MAPK/extracellular signal-regulated kinases (ERK) 1/2 [27,28,29,30,31,32,39], Toll-like receptor 4 (TLR4)/ERK [40,41,42,43,59], phosphatidylinositol-4,5-bisphosphate 3-kinase (PI3K)/protein kinase B (Akt) [26,31,38,49], adenyl cyclase (AC)/cyclic-adenosine mono-phosphate (cAMP)/protein kinase A (PKA) [25,31,44,47,50,51,52], apoptosis signal-regulating kinase 1 (ASK1)-c-Jun amino-terminal kinases (JNK)/p38 [7,33,34,35,36,37,38,39,44,47,48,63], and downstream cAMP response element-binding protein (CREB), JNK [7,24,54,55,56,57,58,59,60,61,63], mammalian target of rapamycin (mTOR) [26,44,45,46], nuclear factor kappa-light-chain-enhancer of activated B cells (NF-κB) [41,42,43,44,47,62], and B-cell lymphoma 2 (Bcl-2)/ Bcl-2 associated X (Bax) balance [57,63,64,65,66,67,68,69]. While a growing understanding of the pathophysiology underlying several nervous system diseases shows that it is related to inflammation and the impairment of cell regeneration, the relationship between the therapeutic mechanism of acupuncture and the p38 MAPK signal pathway has yet to be elucidated. A reverse of the detrimental effect of cerebral ischemic or hemorrhagic injury involves the modulation of the ERK/JNK/p38 signal pathway, which leads to anti-apoptosis of the affected brain area. Improvements of Alzheimer’s disease, vascular dementia, and Parkinson’s disease involves depression or inactivation of the p38 MAPK pathway [32,69,70,71,72,73,74,75,76,77,78,79]. The inactivation of interleukin (IL) 1 β (IL-1β)/p38 in the frontal lobe and hippocampus has a positive effect on improving cognition and memory. Moreover, acupuncture exerts analgesic effects through the interference of both the ascending and descending pain signaling pathway. These findings continue to shed light on the pivot role of the p38 signaling pathway in several nervous system diseases. The common signal transduction pathways through which acupuncture treats nervous system diseases are summarized in Figure 1.

## 2. The p38 MAPKs

MAPKs are a large group of evolutionarily conserved proteins in the plant and animal kingdoms. MAPKs have been implicated in diverse cellular processes including cell survival, proliferation, differentiation, and migration. 

**Figure 2:**
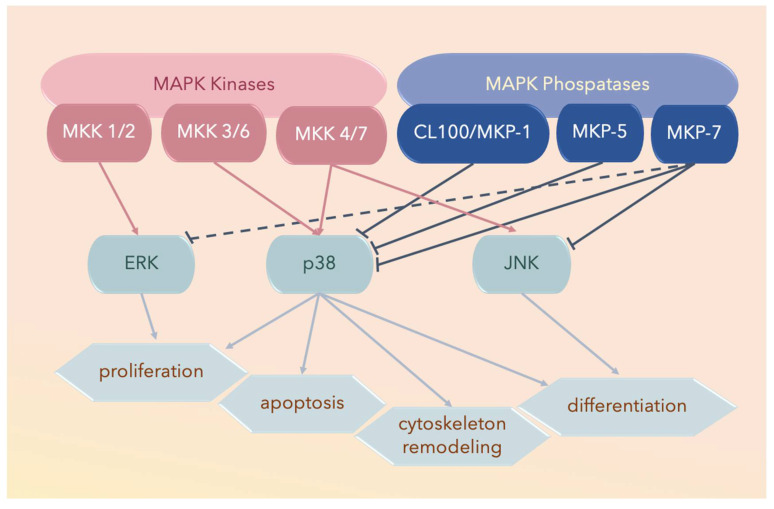
Three major subfamilies of mitogen-activated protein kinases (MAPKs) include extracellular signal-regulated kinases (ERKs), the c-Jun amino-terminal kinases (JNKs), and the p38 MAPKs. The solid lines ending with arrowheads denote activated proteins, solid lines with blunt ends denote deactivated proteins, and the dotted lines with blunt ends denote partially deactivated proteins.

Three major subfamilies of MAPK proteins have been defined: ERK, JNK, and the p38 MAPKs (Figure 2). The middle amino acid residue of the conserved Thr-Xxx-Tyr dual-phosphorylation domain (*dP-consensus*) designates a MAPK protein to its cognate subfamily, and the p38 MAPK subfamily bears the Thr-Gly-Tyr (TGY) dual-phosphorylation domain [77,78].

The most extensively studied MAPK pathways are activated by dual-specificity serine-threonine/tyrosine kinases (STK) termed MAPK kinases (MKKs, MAPKKs, or MAP2Ks), which are activated by MAPKK kinases (MAPKKKs or MAP3Ks). For the p38 MAPK pathway, MKK3 and MKK6 serve as MKKs, and the identified MAPKKKs include MAPKKK2, MAPKKK3, ASK-1, tumor progression locus-2 (Tpl2), and transforming growth factor-β-activated kinase 1 (TAK-1) of the non-canonical transforming growth factor-β (TGFβ) pathway. In general, ERK proteins are primarily activated by growth factors; JNKs are activated by stress-, differentiation-, and growth-related factors; and p38 is activated by stress-related factors [78,79,80,81,82,83,84]. 

The p38 MAPK family comprises p38α, p38β, p38γ, and p38δ (summarized in Table 1). Considering its central role in developmental programming, cellular adaptation to environmental stress, immune responses, inflammation, tissue regeneration, and tumorigenesis, this protein subfamily has gained increasing attention since its initial discovery. Recent studies using new genetic and pharmacological tools have provided essential information regarding the functions of p38 MAPKs in the pathogenesis of prevalent conditions associated with inflammation, diabetes, neurodegeneration, and cancer [78,79,80,81,82,83,84,85,86,87,88,89,90,91,92,93,94,95,96,97,98,99].

The subfamily p38α is reportedly a homolog of *Saccharomyces cerevisiae* Hog1, which is an important regulator of osmotic response [77,78,84,100,101,102,103,104,105]. Other p38 MAPK family members, sharing approximately 60% sequence similarity with p38α, were subsequently cloned and named p38β, p38γ, and p38δ [77,78,84,100,101,102,103,104,105].

**Figure 3:**
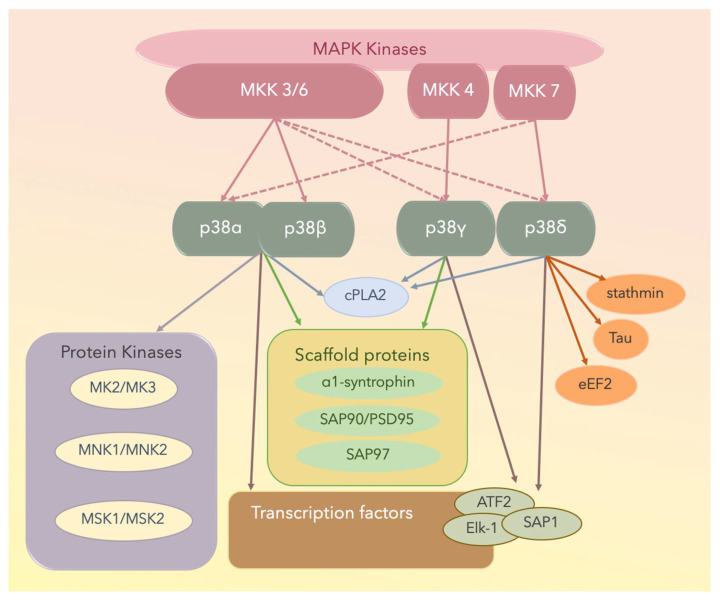
The four p38 MAPK isoforms are p38α, p38β, p38γ, and p38δ. Solid lines denote activated proteins, and dotted lines denote partially activated proteins. MNK: mitogen-activated protein kinase-interacting protein; MSK: mitogen- and stress-activated protein kinase; cPLA2: cytosolic phospholipase A2; ATF2: the activating transcription factor 2; Elk-1: the [erythroblast transformation specific (ETS)] like-1 protein; SAP1: stress-activated protein 1; PSD: postsynaptic density proteins; eEF2: eukaryotic elongation factor 2.

X-ray crystallographic studies have yielded structural insights into the mechanisms underlying the interaction between an MAPKK and an MAPK [103,107]. Both p38α and p38β are well-conserved at both the gene and protein levels and are important in eliciting innate immunity [77,78,84,101,102,103,104,105]. Some p38α and p38β physiological substrates are summarized in Figure 3; these include transcription factors, transcription factor kinases, cytoskeletal proteins, translational machinery components, and other proteins including metabolic enzymes, glycogen synthase, or cytosolic phospholipase A2 (cPLA2). The p38γ and p38δ MAPK isoforms can phosphorylate typical p38 MAPK substrates including transcription factors activating transcription factor 2 (ATF2), erythroblast transformation specific (ETS) like-1 protein (Elk-1), or stress-activated protein kinase (SAP1). However, they cannot phosphorylate MK2 or MK3, which are suitable substrates for the other two p38 MAPK isoforms [109,110,111].

Of the MAPKs, p38γ has a unique short C-terminal sequence, KETXL, which is ideal for binding PDZ domains in proteins, thus accounting for the regulatory role of p38γ in the localization of cellular elements and interactions with cytoskeletal components. Under stress conditions, p38γ interacts with and activates various scaffold proteins including α1-syntrophin, SAP90/PSD95, and SAP97, which are generally targeted to the plasma membrane cytoskeleton at specialized sites, including the neuromuscular junction and gap junctions through protein–protein interactions [111].

With respect to p38δ, it potentially contributes to cytoskeletal regulation because it phosphorylates the cytoplasmic protein stathmin [110,111], which is a crucial regulator of microtubule dynamics and the cell cycle, by promoting the depolymerization of microtubules and/or preventing the polymerization of tubulin heterodimers [109,111]. Furthermore, eukaryotic elongation factor 2 (eEF2) kinase and microtubule-associated protein tau are substrates of p38δ [109,111]. The four p38 isoforms and the substrates are shown in Figure 3.

These four p38 MAPKs are encoded by different genes and display different histotypic expression patterns, with p38α being ubiquitously expressed at significant levels in most cell types, whereas the others potentially display more histotypic expression patterns. For example, p38β is expressed in the brain, p38γ is expressed in skeletal muscle (neuromuscular junction, gap junction), and p38δ is expressed in endocrine glands [108,109,110,111,112,113,114,115]. The genetic ablation of specific p38 MAPK family members has revealed functional redundancy in this protein subfamily. For example, the osmotic shock-induced phosphorylation of the stress-activated protein 97 (alternatively termed synapse-associated protein 97, SAP97; also known as discs large homolog 1 scaffold protein, hDLG1) is usually mediated by p38γ; however, in the absence of this kinase, other p38 MAPKs can perform this function.

## 3. MAPK Substrates, Signaling Pathways, and Functions

Nine members of the dual-specificity phosphatases specific for MAPKs, termed MKPs, have been reported. Each member has specific substrates, tissue distribution, and subcellular localization. For example, MAPK phosphatase 7 (MKP-7) binds to and inactivates p38α and p38β through dephosphorylation; however, it does not interact with p38γ and p38δ; MKP-5 and CL100/MKP-1 also bind to p38α and p38β, but not to p38γ or p38δ [115] (Figure 2 and Figure 3).

Several studies have revealed a direct interaction of the N-terminal region of the MAPKK with a docking groove present on the surface of the MAPK distant from the catalytic active site. The second determinant of MAPKK specificity is the structure of the MAPK activation loop harboring the Thr-Xxx-Tyr dual-phosphorylation motif. The specificity of these interactions partly mediates the potential of an individual MAPKK to selectively activate a particular MAPK. 

A recent study emphasized that dynamic changes are necessary for enzyme activity [116]. For instance, two MAPKs, ERK2 and p38, are differentially activated owing to differences in dynamism. A comparison of the dynamics of PKA and p38 revealed similarities in their dynamic properties.

### 3.1. Dual Phosphorylation by MKKs

In yeast, only a single MAPKK appears to activate each MAPK, whereas mammalian MAPK signaling modules include more than one MAPKK. The MAPKKs responsible for activating the p38 MAPK pathways appear to be specific to cell type and stimulus [105,107,114,116]. Brancho and Tanaka et al. investigated the mechanism underlying p38 activation in vivo by examining the effect of disruption of the murine *Mkk3*, *Mkk4*, and *Mkk6* genes on the p38 MAPK signaling pathway [100]; they found that Mkk3 and Mkk6 are essential for tumor necrosis factor (TNF)-mediated p38 activation. By contrast, ultraviolet (UV) radiation-induced p38 activation was mediated by Mkk3, Mkk4, and Mkk6. Furthermore, they reported that the role of Mkk4 in p38 MAPK activation in fibroblasts was largely redundant with those of Mkk3 and Mkk6. Mkk4 is a potentially important p38 MAPK activator in cells with low levels of Mkk3 and Mkk6. These data indicated that p38 MAPK was regulated by the coordinated and selective action of the three different protein kinases MKK3, MKK4, and MKK6 in response to cytokines and exposure to environmental stress. The inactivation of p38 MAPK was reportedly associated with defects in cell cycle arrest and increased tumorigenesis [117,118,119,120,121,122,123].

**Figure 4:**
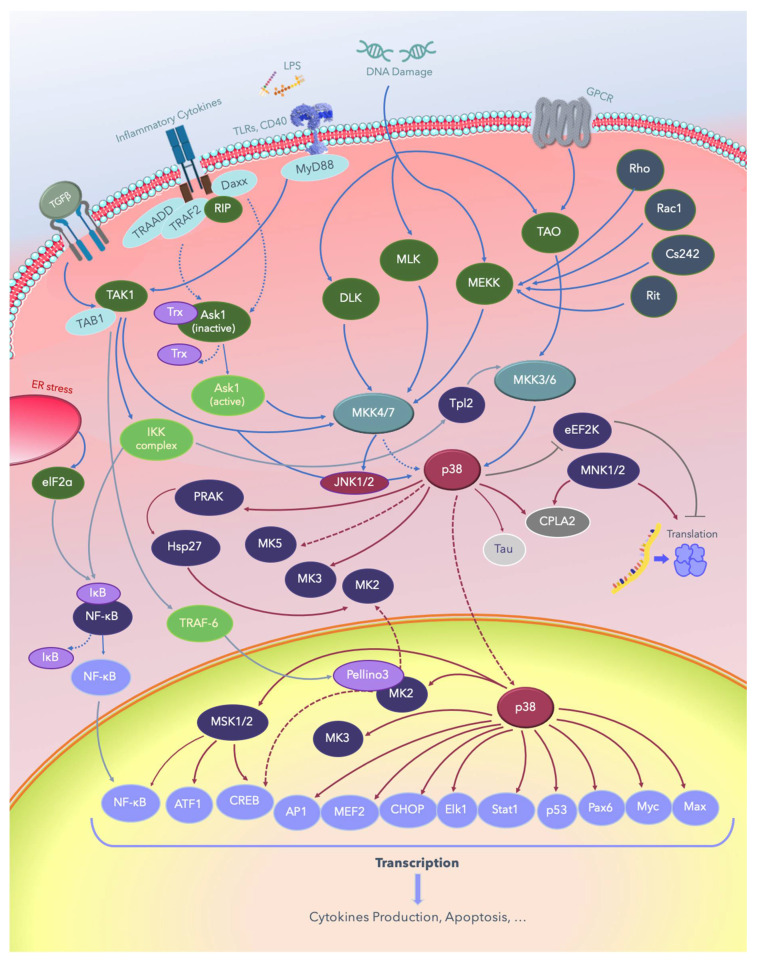
The p38 MAPK signaling pathways. Solid lines indicate signaling pathways and the proteins involved in them; dotted lines indicate the regulatory mechanisms reported in several studies, and lines with blunt ends indicate pathways inhibiting or deactivating downstream substrates. LPS: lipopolysaccharide; TGFβ: growth factor beta; TLRs: Toll-like receptors; CD40: cluster of differentiation 40 receptors; GPCRs: G-protein-coupled receptors; elF2a: eukaryotic translation initiation factor 2A; ER: endoplasmic reticulum; NF-κB: nuclear factor kappa B; TAB1: TGF-beta-activated kinase1; TRADD: tumor necrosis factor receptor type 1-associated DEATH domain protein; TRAF: tumor necrosis factor receptor (TNFR)-associated factor; Daxx: death domain-associated protein; RIP: receptor-interacting protein kinases; MyD88: myeloid differentiation primary response 88; PRAK: p38-regulated and -activated kinase; Hsp: heat shock proteins; DLK: dual leucine zipper kinase; MLK: mixed-lineage protein kinase; MEKK: MEKK kinase; MEK: MEK kinase; MKK: MK kinase; MNK: mitogen-activated protein kinase-interacting protein; TAO: thousand and one amino acids; eEF2K: eukaryotic elongation factor 2 kinase; cPLA2, cytosolic phospholipase A2; ATF: activating transcription factor; MSK: mitogen- and stress-activated protein kinase; CREB: cAMP response element–binding protein; AP-1: activator protein 1; MEF: myocyte enhancing factor; CHOP: C/EBP homologous protein, a member of the CCAAT/enhancer-binding proteins; Elk-1: erythroblast transformation specific (ETS) like-1 protein; Stat1: signal transducer and activator of transcription 1; Pax6: a paired-box protein encoded by the master gene Pax-6; Myc: a group of transcription factors coded by a regulator genes and proto-oncogene called Myc; Max: a transcription factor coded by the myc-associated factor X.

Several MAPKKKs have been implicated in the regulation of p38 MAPK signaling, including the mixed-lineage kinases (MLKs), apoptosis signal-regulating kinase 1 (ASK-1), TAK-1, and some members of the MAPK/ERK kinase kinase (MEKK) family. Low-molecular-weight guanosine-5′-triphosphate (GTP)-binding proteins of the Rho subfamily, including Ras-related C3 botulinum toxin substrate 1 (Rac1), the erythrocyte membrane glycoprotein Cd242, the nonlipid modified Ras-related protein (Rit), the transcription termination factor Rho, and heterotrimeric G-protein-coupled receptors (GPCRs) contribute to p38 activation upstream of MAPKKKs [124,125,126,127,128,129,130,131,132,133,134,135]. More recently, a new signaling pathway, different from TAK1, that involves the inhibitor of nuclear factor kappa-B kinase (IκBK), NFκB/p105, and Tpl2 stimulating MKK3/6, and downstream p38 was established in macrophages [135,136,137,138,139]. The p38 MAPK pathways are shown in Figure 4.

### 3.2. Autophosphorylation

**Figure 5:**
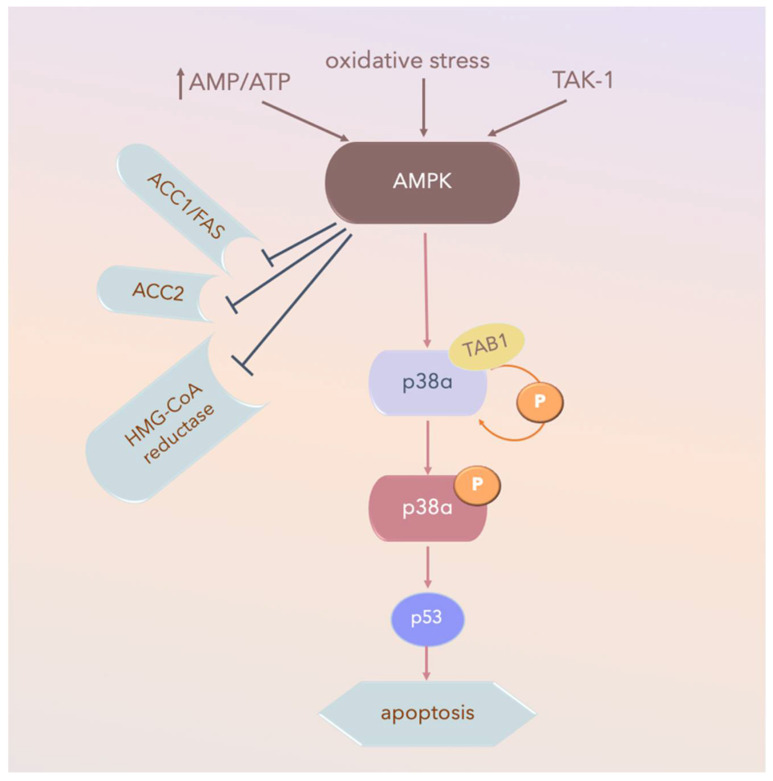
AMPK-activated-p38α pathways in ischemic heart tissue. Arrow: activated; TAK-1: transforming growth factor-β-activated kinase 1; AMPK: 5’ AMP-activated protein kinase; P: phospho-; AMP: adenosine mono-phosphate; ATP: adenosine tri-phosphate; AMP/ATP: AMP/ATP ratio; ACC1: Acetyl-CoA carboxylase 1; Fas signal pathway, Fas and Fas Ligand (FasL) regulate cell death; HMG-CoA reductase: a rate-controlling enzyme of the mevalonate pathway responsible for cholesterol and other isoprenoid biosynthesis.

MKK-independent activation is achieved through autophosphorylation and activation of p38α after interaction with TGFβ-activated kinase 1 (TAB1), which appears to activate p38α via the 5’ AMP-activated protein kinase (AMPK) in ischemic heart tissue (Figure 5). TAB1 sequesters p38α in the cytosol, thus potentially preventing some MKK-activated p38α functions. However, this mechanism does not contribute to p38 MAPK activation in fibroblasts or epithelial cells under the same conditions [134].

Another MKK-independent mechanism underlying p38α activation has been observed in T-cell stimulation, wherein p38α is activated by T-cell antigen receptor (TCR)-mediated stimulation through p38α phosphorylation on a noncanonical activating residue, Tyr323. This activated p38α alters its structural conformation, phosphorylating third-party substrates and its TGY motif [115].

## 4. TRPV1 and the p38 Pathway

Transient receptor potential vanilloid receptors (TRPVs) are mechanosensitive channels highly associated with nervous system functions including pain, memory, and mechanical sensations. Furthermore, capsaicin receptor TRPV1 is a key regulator of pain and inflammation and is upregulated in microglia in the brain, especially in the anterior cingulate cortex [138,139,140]. The stimulation of microglial TRPV1 induces cortical microglial activation and indirectly enhances glutamatergic neuronal transmission by promoting the shedding of extracellular microglial microvesicles (release of vesicles in the extracellular space [141]). Moreover, in the cortex of mice with neuropathic pain, TRPV1 affects the intrinsic electrical properties of neurons and synaptic strength [142,143]. This signal transduction can be inhibited by the p38 MAPK inhibitor SB203580. Thus, p38 MAPK is a downstream TRPV1 activator whose phosphorylation plays an essential role in microglial microvesicle shedding by activating P2X purinoceptor 7 (P2X7) ATP receptor [138,139,140,141,142,143]. Whereas TRPV1 mediates communication between microglia and neurons, inhibition of the phosphorylation of its downstream p38 MAPK inhibits sphingosine metabolism [140,141,142,143].

Furthermore, several studies have examined biomarkers of nerve damage, including astrocytic marker glial fibrillary acidic protein (GFAP), microglial marker ionized calcium-binding adapter molecule 1 (Iba-1), S100 calcium-binding protein B (S100B), and the receptor for advanced glycation end-products (RAGE), revealing marked upregulation of these molecules in the dorsal root ganglion (DRG) and spinal cord dorsal horn (SCDH) of Complete Freund’s adjuvant (CFA)-treated mice. This inflammatory effect was reversed through electroacupuncture, which achieved an equivalent result to that of TRV1 gene deletion [143].

## 5. Brain-Derived Neurotrophic Factor and p38 Pathways

Microglia cells are resident macrophages in the central nervous system (CNS) with a small soma with thin and branched processes. In the case of neural injury, their processes rapidly migrate toward the site of injury. In peripheral nerve injury, spinal cord microglia are activated. Spinal P2X4 receptors (P2X4Rs), phosphorylated p38 MAPK (p-p38-MAPK), and brain-derived neurotrophic factor (BDNF) are upregulated in spared nerve injury (SNI) rats [144]. BDNF signals to neurons in spinal lamina I and increases intracellular chloride concentration, thereby counteracting gamma-aminobutyric acid (GABA)- and glycine-mediated inhibition in these cells. The disinhibition unmasks innocuous inputs to lamina I neurons and facilitates their responses to noxious inputs [145]. In other studies, BDNF/TrkB was reported to promote inflammation in spinal cord injury through the p38 signaling pathway in the murine model [40,146]. Moreover, an upregulation of BDNF expression was observed in rats when applying electroacupuncture (EA) to acupoints GV20 and GV14, which later revealed that EA counteract caspase-3-dependent neuronal apoptosis by activating the Raf-1/MEK1/2/ERK1/2/p90RSK/Bad signaling pathway [147]. 

These results suggest that increasing the expression of BDNF synthesis, the downregulation of P2X4Rs, and the inhibition of p38 phosphorylation might account for the therapeutic effects induced by acupuncture in nerves injury. 

## 6. Acupuncture and the Effects of Electric Fields on Nerve Regeneration

Previous in vitro and a few in vivo studies have reported positive nerve growth-promoting effects of electric fields where the cathode was placed toward the distal end of the injured nerve stumps [148,149,150,151,152,153,154]. Chen et al. reported that regenerated nerves in the electrical stimulation group may have a more mature ultrastructure compared with those in the control group [155]. Furthermore, they reported low regeneration success in patients receiving electrical stimulation relative to controls [155].

Together with several previous clinical studies [155,156,157,158], these studies show that patients with nerve anastomosis should not receive any electrical stimulation for rehabilitation until their reconnected nerve stumps have grown into a more mature stage of regeneration. One of the mechanisms underlying electroacupuncture (EA) is the relief of acute pain through the release of opiates to activate μ-, δ-, and κ-opioid receptors; furthermore, it can regulate persistent pain by activating μ- and δ-opioid receptors [25]. These findings explain the application of EA in treating neuropathic pain when pharmacotherapeutic approaches are ineffective.

Furthermore, the analgesic effect of low-frequency (2 Hz) EA is exerted on the noradrenergic descending pathway, involving the modulation of spinal GABAergic nerves (GABA_A_). Administering EA at 2 Hz at acupoint ST36-37 reportedly relieved neuropathic pain through long-term depression (LTD) of the C-fiber [159]. This phenomenon could be blocked by N-methyl-D-aspartate (NMDA) and an opioid receptor antagonist. By contrast, high-frequency (100 Hz) EA induces long-term potentiation (LTP) of endogenous GABAergic (GABA_B_) and the serotonergic inhibitory systems through modulation of μ-opioid and 5-hydroxytryptamine 1 (5-HT1) receptors [25,160]. Other studies have reported that delivering EA at acupoints GV14 and GV20 exerts neuroprotective effects by activating CREB, BDNF [161], and α7 nicotinic acetylcholine receptor (α7 nAChR) [162] while simultaneously reducing S100B-mediated neurotoxicity [163]. Moreover, EA at acupoint ST36 can evoke excitatory signals in either the peripheral nervous system or CNS in vivo [163].

## 7. Inflammatory and Neuropathic Pain

### 7.1. Inflammatory Pain

Protein kinase C (PKC) is rapidly activated by heat or bradykinin and translocates, assisted by scaffolding proteins, from intracellular compartments to the plasma membrane. In rat DRG, the TRPV1 signaling pathways involve the phosphatidylinositol 3-kinases (PI3K), PKC, and calmodulin-dependent protein kinase II (CaMKII) [143,163]. The p38α MAPK was first recognized for its role in inflammation where it regulates the biosynthesis of proinflammatory cytokines IL-1 and TNFα [77,78,98,99,100,101,102,103,104,105,115,164,165,166,167,168,169]. After noxious stimulation, activated p38 (phospho-p38) is increased in the spinal cord and DRG neurons and continues to propagate pain signaling by phosphorylating transcription factors and proinflammatory cytokines including TNF-α [168,169]. Inflammation is induced through the activation of these cytokines, followed by their interactions with their cognate receptors and via the small GTPases (e.g., Rac1 and CDC42), leading to p38 activation [169].

### 7.2. Neuropathic Pain

In contrast to physiological pain, pathological pain does not depend on the presence of tissue-damaging stimuli. Neuropathic pain can be agonizing, potentially persisting over long periods, and it is often resistant to known painkillers. Increasing evidence indicates that spinal microglia react and undergo a series of changes that directly influence the establishment of painful peripheral neuropathy [168,169,170]. After nerve damage, purinergic P2X4 receptors (nonselective cation channels activated by extracellular ATP) are upregulated in spinal microglia in a manner dependent on transcription factors interferon regulatory factor 8 and 5 (IRF8 and IRF5), both of which are expressed in microglia after peripheral nerve injury [171,172]. Furthermore, in spinal microglia, the response to extracellular stimuli results in signal transduction through intracellular signaling cascades involving p38 and ERK. Inhibition of the function or expression of these microglial molecules suppresses the aberrant excitability of dorsal horn neurons and neuropathic pain [172].

**Figure 6:**
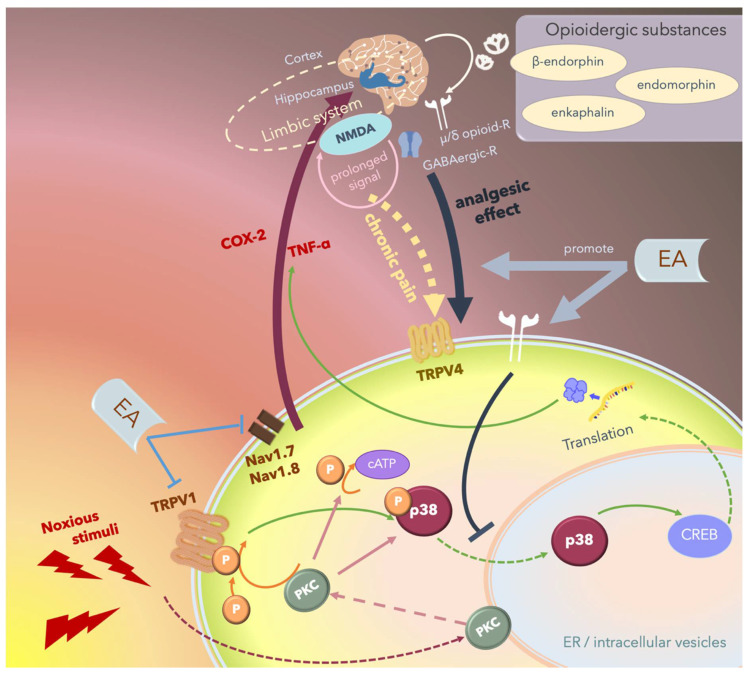
Schematic of the potential mechanism underlying the electroacupuncture (EA)-mediated analgesic effect. Noxious stimuli including heat or proinflammatory cytokines are transduced by the transient receptor potential vanilloid 1 (TRPV1) into the cells, thus phosphorylating and activating p38, which translocates from the cytosol to the nucleus and promotes transcription by affecting cAMP response element-binding protein (CREB), thus upregulating specific proteins including inflammatory cytokines or inducing apoptosis. These noxious stimuli can also directly activate protein kinase C (PKC). Nociception regulation by TRPV1 and Na_v_1.7/1.8 receptor stimulation in the peripheral nerves propagates the signal through the ascending pathway and upregulates proinflammatory cytokines including cyclooxygenase-2 (COX-2) and tumor necrosis factor-α (TNFα) in the central nervous system, and the signal is then perceived as “pain”. Chronic pain re-emerges when nociceptive signals cause prolonged stimulation through hippocampal N-methyl-D-aspartate receptor (NMDA) receptors and some other areas of the limbic system, and the signals are transmitted through the descending pathway, stimulating TRPV4 and triggering pain. Endogenic analgesic mechanisms involve the release of opioidergic substances that bind to γ-aminobutyric acidergic (GABAergic) receptors and μ- or δ-opioid receptors that act locally in the central nervous system and inhibit the descending pain pathway. EA exerts therapeutic effects by inhibiting the ascending pain pathway and intracellular p38-mediated inflammatory pathway by stimulating peripheral opioid receptors. Furthermore, EA promotes endogenic analgesic mechanisms, thus exerting immediate local analgesic effects and rescuing CNS-induced chronic pain.

Since MAPKs significantly contribute to the development of hyperalgesia, the inhibition of any of the three pathways, namely the ERK, p38, and JNK pathways, can rescue inflammatory or neuropathic pain. Several studies have reported that 2- and 15-Hz EA can downregulate cerebral TRPV4 expression and attenuate chronic constriction injury (CCI)-induced neuropathic pain in an animal model [173,174,175,176,177,178,179]. Furthermore, Huang et al. reported that EA modulates both excitatory and inhibitory neurotransmitters to relieve neuropathic pain in the higher brain regions [175]. The hippocampus plays an integral role in the transition from acute to chronic pain in the limbic system by activating NMDA receptors and subsequently prolonging acute nociceptive stimuli that continue activating the descending pathways of pain (Figure 6).

### 7.3. Post-Operation Pain

Presurgical sham-, low-, and high-frequency EA treatments were reported to significantly reduce the postoperative patient-controlled analgesia morphine requirement; moreover, both low- and high-frequency EA decreased opioid-related side effects including nausea and dizziness throughout the first postoperative day [176,177]. Among these studies, ST36 and LI4 were the most frequent acupoints manipulated to achieve analgesic effects [summarized in Table 2].

Furthermore, overexpression of the voltage-gated sodium channel (Na_v_), TRPV1, and acid-sensing ion channel 3 (ASIC3) in DRG neurons in response to proinflammatory cytokines is a key component of inflammatory pain [179,180,181,182,183,184,185,186,187,188,189,190,191,192]. Low-frequency (2-Hz) EA at acupoint ST36 can reduce inflammatory pain by attenuating ASIC3 overexpression in peripheral DRG neurons. ASIC3 downregulation potentially prolongs the clinical benefits of EA. Goldman et al. reported that acupuncture stimulates adenosine A1 receptor and induces adenosine release, thus relieving inflammation and neuropathic pain, which was not observed in mice lacking adenosine A1 receptors [193]. These results suggest that the opioid and adenosine pathways potentially contribute to analgesia in both manual acupuncture and EA.

### 7.4. Migraine

Migraine is a complex disorder; each episode begins with prodromes and aura (transient focal neurological symptoms). The origin of recurrent headache accompanied by visual or sensory symptoms is speculated to involve the hypothalamus, brain stem, and cortex. Current theories suggest that migraine is a neurovascular disorder involving cortical spreading depression, neurogenic inflammation, and vasodilation [194,195]. Owing to the disease itself or its genetic underpinnings, the brains of individuals who experience migraine are altered structurally and functionally; these molecular, anatomical, and functional abnormalities provide a neuronal substrate for extreme sensitivity to fluctuations in homeostasis, decreased adaptability, and recurrent headaches [194]. 

A randomized controlled trial investigated the efficacy and tolerability of acupuncture in comparison with topiramate treatment in chronic migraine prophylaxis. The effectiveness of acupuncture was similar to or greater than that of prophylactic pharmacotherapy, with fewer side effects in migraine [194]. The selected acupoints were bilateral BL2, GB20, EX-HN5, and EX-HN3, which are all associated with the trigeminal and cervical dermatomes. Although different combinations of peripheral effects, spinal/supraspinal mechanisms, and cortical and psychological mechanisms potentially contribute to the clinical effects of acupuncture, several other studies have reported that acupuncture potentially exerts curative effects not only through local analgesia but also through anti-inflammation, neuropeptide regulation, cytoskeleton remodeling, cell repair, and improvement in overall homeostasis.

### 7.5. Transcutaneous Electrical Nerve Stimulation Versus EA

The difference between transcutaneous electrical nerve stimulation (TENS) and EA is that TENS involves the application of the cathode at the affected area rather than at acupoints. Furthermore, TENS is used to reduce mechanical hyperalgesia in knee joint inflammatory pain [196,197], although its effectiveness is only sustained for a short time (several days) [198,199]. The analgesic effect of low-frequency TENS and EA can be attenuated using μ-opioid receptor blockers, thus indicating the involvement of peripheral opiate release in the underlying mechanism [196]; furthermore, the blockade of β-endorphin and corticotropin-releasing factor also reduces EA-induced analgesia.

### 7.6. Hypothesis Regarding the Long-Term Effect of Acupuncture

Although proinflammatory cytokines are regulated at different levels, the role of the p38α MAPK pathway in posttranscriptional regulation has received increasing attention. Numerous p38 MAPK–regulated mRNAs often contain an adenylate-uridylate-rich element (termed AU-rich element or ARE) in the 3′ untranslated region (3′UTR) [200,201]. The regulation of mRNA stability is particularly important for the expression of proteins involved in inflammatory response. These elements target mRNAs for rapid decay. The decay of ARE-containing reporter mRNAs is blocked upon p38 MAPK activation. Numerous ARE-containing endogenous mRNAs of inflammatory proteins are destabilized upon p38 inhibition. Some studies have reported a common mechanism underlying gene regulation via the p38α MAPK pathway for posttranscriptional regulation by the ARE. 

These results are consistent with those of several previous animal and clinical studies reporting that EA improves analgesia during the acute phase posttraumatic healing and pain after major surgery [176,177]. Numerous prospective randomized controlled trials on carpal tunnel syndrome (CTS) have reported that acupuncture at acupoints PC7 and PC6 is as effective and safe as steroid treatment of mild-to-moderate CTS, both in the acute phase and throughout the 1-year follow-up period [187,188]. Based on the analgesic mechanisms of acupuncture and EA, which attenuate the p–p38 signaling pathway, the long-term effect of acupuncture or EA may result from neural modulation through posttranscriptional regulation. The potential mechanism underlying EA-mediated analgesic effects is depicted in Figure 6. The underlying mechanisms and the primary results of these studies are summarized in Table 2. 

## 8. Cerebral Ischemia

During a cerebral ischemia (CI) event, hypoxia is induced in the brain, followed by the upregulation of various cytokines and potentially neurotoxic molecules. Nitric oxide reportedly influences cerebral oxygen vasoreactivity during severe hypoxia [202]. Both manual acupuncture and EA can increase cerebral blood flow [13,14]. Furthermore, 2- and 15-Hz EA at acupoint ST36 increased cerebral blood fluid (CBF) in rats with and without CI [14]. Per the traditional Chinese medicine theory, the Governor Vessel (or the Governor Meridian) directly communicates with the brain. In a previous study, EA at acupoints GV20 and GV14 before artificial mild cerebral ischemia–reperfusion (I/R) injury in an animal model exerted a neuroprotective effect against reperfusion injury [165]. Another neuroprotective mechanism of EA involves the downregulation of astrocytic S100B, which in turn deactivates p38 MAPK and its downstream substrate NF-κB. These effects subsequently reduce oxidative/nitrative stress and inhibit the TNF-α/tumor necrosis factor receptor type 1-associated DEATH domain protein (TRADD)/ Fas-associated protein with death domain (FADD)/cleaved caspase-8/cleaved caspase-3 apoptotic pathway in the ischemic cortical penumbra after reperfusion [89,201,202,203,204,205,206,207]. Both 5- and 25-Hz EA at acupoints GV20 and GV16 effectively downregulated reactive astrocytosis to exert neuroprotective effects against cerebral infarction-induced neuronal apoptosis, probably by activating the p38 MAPK/CREB signaling pathway [203].

Heat shock proteins (Hsp) significantly contribute to cellular regenerative processes in injured tissues because Hsp is upregulated during stem cell differentiation; however, the depletion or inhibition of Hsp70/Hsc70 impairs this process [206]. Hsp27 and Hsp70 reportedly contribute to intracellular protein repair after acute CI [206,207,208,209,210,211]. The dual phosphorylation of p38 MAPK was increased through early ischemia in an in vitro study wherein Hsp27 served as a terminal substrate of the p38 MAPK cascade. A later study indicated that among the p38 MAPKs, p38γ is the principal isoform responsible for the phosphorylation of HSF1 at Ser326 (S326) in cells (phosphorylation at S326 is a hallmark for HSF1 activation) [212], thus contributing to mitotic progression. A protease–mass spectrometry approach unexpectedly revealed that p38 MAPK also catalyzes the phosphorylation of HSF1 at S303/307, which is a repressive posttranslational modification [212,213]. Hsp70 bound MK2 to regulate MK2–p38MAPK interaction in the stem cells, and the essential regions required for Hsp70–MK2 interaction have been identified [206,209,210,211,212,213]. Taken together, Hsp can regulate cell differentiation by interacting with MK2 to stabilize p38 MAPK.

Another study administered 2 Hz EA the GV20 and ST36 acupoints in rat models of cerebral I/R injury and observed lowered peak levels of adrenocorticotrophic hormone and HSP70, suggesting that EA may inhibit excessive stress, reduce inflammation, and promote neural repair, thus facilitating healing in ischemic stroke [211]. The mechanisms and main results of the identified articles are summarized in Table 3.

## 9. Epilepsy and Seizure

Epilepsy is characterized by an abnormal electric discharge of the brain, especially in the hippocampus. Epilepsy is usually associated with neuronal loss, astrocyte proliferation, mossy fiber sprouting, and synaptic reorganization in the hippocampus [40,214,215,216,217,218,219]. Pharmacological treatment approaches, including those employing Western medicine and Chinese herbal medicine, have been attempted to treat or prevent the harmful effect of seizures. For example, *Uncaria rhynchophylla* is a herb used in traditional Chinese medicine for epilepsy management; its therapeutic effect was reportedly associated with attenuated mossy fiber sprouting, astrocyte proliferation, S100B protein overexpression, and increased hippocampal neuronal survival [219].

Acupuncture has been applied for clinically managing epilepsy. Previous studies have reported that auricular acupuncture positively influences drug-resistant epilepsy patients, potentially through vagus nerve stimulation, attenuating the hyperactive hippocampus, regulating inflammatory cytokine pathways, protecting the hippocampus from apoptosis, and ameliorating the sprouting of mossy fibers in the hippocampus [40,218,220,221]. Several antiepileptic mechanisms have been proposed, including the downregulation of JNK or ERK1/2 and pro-inflammatory factors (IL-1β, IL-6, TNF-α) in the cerebral cortex and hippocampus [216]. Another study suggested that auricular EA and EA at ST36-ST37 achieved therapeutic effects by reducing hippocampal hyperactivity and the transient receptor potential cation channel subfamily A member 1 (TRPA1), PKCε, PKCα, and pERK1/2 signaling pathways [40]. Moreover, auricular EA with EA at ST36–ST37 exerted long-term (6-week observation in the study) beneficial effects by reducing the anti-inflammatory response in numerous cyclooxygenase-2 (COX-2) immunoreactive cells and hippocampal COX-2 levels [218].

Acupuncture exerts antiepileptic effects by inducing anatomical changes and changes in neurotransmitter, inflammatory cytokine, and transcription factor levels. Regarding studies to date, the related mechanisms primarily involve the suppression of TRPA1/pERK and TLR4/ERK pathways and activation of the PI3K/Akt pathway. Although some studies have reported the inactivation of the TLR4 pathway, accompanied by a reduction in pCaMKIIα, pERK, pp38, pJNK, and pNFκB levels [40], the role of p38 and its association with the aforementioned signaling pathways remain unclear. These mechanisms and the primary results of the aforementioned studies are summarized in Table 4.

## 10. Motion Sickness

External noxious stimulation alters intracellular signal transduction pathways in primary afferents, potentially contributing to pain hypersensitivity. This pathway proceeds via TRPVs, especially TRPV1 and TRPV4, leading to the rapid phosphorylation of p38 MAPK in the afferent neurons and the induction of hyperalgesia. Recent studies have revealed that thermal stimulation may not be the only trigger for TRPV activation [222,223]. Kaolin consumption was quantified as a behavioral response of an emetic reflex (pica behavior) in a murine model. A previous study reported that TRPV1 levels were significantly increased upon stimulation of motion sickness, and EA at PC6 acupoints attenuated hypothalamic TRPV1 levels and exerted an antiemetic effect [222]. The acupoint PC6 is well-known for its therapeutic effect, especially in relieving nausea and vomiting. This study revealed that the mechanism underlying the reduction of motion sickness potentially involves the TRPV1 pathway [222].

## 11. Degenerative Nerve Diseases

Microglia, the endogenous macrophages of the CNS, monitor their territory through a constant movement of their elaborated thin processes and responses to local stressors and immune disruptions. Clinical studies and preclinical animal models have implicated the dysregulation and overproduction of proinflammatory cytokines from activated microglia in the CNS as contributors to the pathophysiology of chronic neurodegenerative disorders including Alzheimer’s disease [16], Parkinson’s disease, and multiple sclerosis and acute neurodegenerative conditions including traumatic brain injury and stroke. The p38α MAPK pathway helps increase the microglial production of proinflammatory cytokines induced by diverse stressors [16,17,223]. These results indicate the feasibility of targeting p38α MAPK to modulate the production of pro-inflammatory cytokines by the CNS.

Few studies have focused on the effects of androgens on neuroinflammation. Yang et al. investigated the neuroprotective role of androgens (including testosterone and its metabolite dihydrotestosterone, DHT) in lipopolysaccharide (LPS)-induced neuroinflammation, neuronal damage, and behavioral dysfunction [18]. DHT potentially inhibits the LPS-induced release of proinflammatory factors in primary microglia by suppressing the TLR4-mediated NF-κB and p38 signaling pathways, thus protecting neurons from inflammatory damage induced by activated microglia.

Sodium ferulate is used in traditional Chinese medicine (such as the root of *Ligusticum chuanxiong* and *Angelica sinensis*) and is speculated to help treat cardiovascular and cerebrovascular diseases by preventing thrombosis. Furthermore, sodium ferulate prevents Aβ-induced MKK3/MKK6-p38-Hsp27 signaling and reduces apoptosis in the rat hippocampus [17].

### 11.1. Alzheimer’s Disease

The pathological hallmarks of Alzheimer’s disease (AD) are the accumulation of extracellular plaques and intracellular neurofibrillary tangles that are composed of filaments of β-amyloid polymers and the neuronal microtubule-associated protein Tau, respectively. Elevated β-amyloid levels in an AD brain are speculated to induce microglial activation and the consequent release of proinflammatory cytokines induced by the p38 MAPK pathway, potentially contributing to AD pathogenesis together with other disorders including neuronal injury, trauma, ischemia, and the accumulation of oxidants associated with brain aging [16,18,224].

Since more than half of the phosphorylation sites in paired helical filament–Tau are serine and threonine residues followed by proline, members of the MAPK family, especially p38α, potentially play an important role in phosphorylating Tau [16]. Considering that aberrantly activated JNK and p38 MAPKs are reportedly associated with cells containing filamentous Tau in some neurodegenerative diseases, these kinases may contribute to Tau hyperphosphorylation. Moreover, the p38 MAPK activator, MKK6, is active in neurodegenerative diseases, indicating a potential contribution to Tau hyperphosphorylation in these diseases. Recent studies have reported that Tau is a suitable in vitro substrate for p38 isoforms p38δ and p38γ, and its phosphorylation by these two enzymes reduces its ability to promote microtubule assembly [18,225,226,227,228]. Moreover, p38γ overexpression in neuroblastoma induces Tau phosphorylation, which is associated with a reduction in Tau that is associated with the cytoskeleton and an increase in soluble Tau. Furthermore, p38δ is the major Tau kinase in neuroblastoma in response to osmotic shock [227]. This evidence indicates that p38 MAPKs potentially regulate Tau hyperphosphorylation in neurodegenerative diseases and are potentially suitable therapeutic targets for those diseases.

Other studies have focused on the receptor for advanced glycation end products (RAGE), a multiligand member of the immunoglobulin superfamily of cell surface molecules, which serves as a receptor for Aβ on neurons, microglia, astrocytes, and endothelial cells of blood vessels. RAGE is upregulated in brain regions affected by AD. A murine transgenic model revealed that RAGE is a potential therapeutic target for AD [229].

Several studies have reported that EA and manual acupuncture have a therapeutic effect on AD. For example, EA at acupoint GV20 suppresses Aβ generation [228]. Stimulation at acupoints GV14 and BL23 downregulates beta-secretase 1, which is an enzyme that is responsible for Aβ generation in AD and increases hippocampal ATP levels in AD mice [230]. Low-frequency (2-Hz) EA at acupoint GV20 and bilateral acupoints KI3 and ST36 for 15 min once daily for 12 sessions was reported to significantly downregulate p-p38 MAPK and IL-1β mRNA expression in the hippocampus and the frontal lobe [65]. Other studies have reported that EA at GV20 reversed behavioral deficit and LTP impairment, possibly through the N-methyl-D-aspartate receptor 1 (NMDA-R1) and TRPV1 pathway, thus reversing NR1- (one of the NMDA receptor subunits) and TRPV1-mediated neurotoxicity in vascular dementia [208,209]. This implies that 2-Hz EA can rescue p38 MAPK signal transduction, resulting in anti-apoptotic and anti-inflammatory effects that reduce Aβ deposits in the brain and improve learning and memory in AD patients [20].

### 11.2. Parkinson’s Disease

Parkinson’s disease is a neurodegenerative disorder that causes severe motor impairment owing to loss of dopaminergic neurons in the substantia nigra pars compacta (SNpc). Several studies have focused on PD pathophysiology and reported potential therapeutic targets for PD.

One of the direct neuroprotective effects is exerted by the protein deglycase DJ-1, which inhibits ASK1 through the nuclear sequestration of death domain–associated protein (Daxx) [230,231,232]. DJ-1 is speculated to scavenge reactive oxidative species (ROS) and subsequently attenuate oxidative stress, thereby reducing cellular ROS burden [233,234]. Furthermore, DJ-1 interacts with p38-regulated/activated kinase (PRAK/MK5) under cellular oxidative stress to facilitate nuclear localization of DJ-1 [235]. Thus, DJ-1 co-localizes with PRAK in the nuclei of NIH3T3 cells. Since DJ-1 lacks both nuclear localization and nuclear export signals, PRAK may be a crucial factor assisting DJ-1 in regulating the cellular localization of Daxx and in ASK1 signaling and concomitant cell death [236].

A recent study reported that EA at acupoint KI3 reduced the excitotoxicity of dopaminergic neurons by regulating the NMDA receptor function and thus may potentially serve as a novel therapeutic approach for PD [237]. The levels of pNR1 and pNR2B, phosphorylated PKA, PKC, α-Ca2+/CaMKII, pERK, and CREB were also reduced following EA [237].

## 12. Fibromyalgia

Fibromyalgia is a common chronic pain syndrome characterized by chronic widespread mechanical pain. TRPV1 and TRPV4 are believed to play a crucial role in the pathophysiology of fibromyalgia. EA at 2 Hz at bilateral ST36 acupoints was reported to reduce long-lasting mechanical hyperalgesia by downregulating TRPV4, p-p38, p-JNK, and pCREB in the peripheral nervous system and CNS [238,239].

Taken together, acupuncture exerts anti-inflammation, anti-apoptosis, and neuroprotection effects via modulating proinflammatory cytokines, increasing the levels of neurotrophic factors and modulating signaling pathways, such as p38 MAPKs, Raf/MAPK/ERK1/2, TLR4/ERK, PI3K/AKT, AC/cAMP/PKA, ASK1–JNK/p38, and downstream CREB, JNK, m-TOR, NF-κB, and Bcl-2/Bax balance. EA relieves acute pain through inhibiting the TRPV1 signaling and PI3K/PKC/CaMKII pathway. EA also promote the release of opiates to activate μ-, δ-, and κ-opioid receptors, therefore producing immediate and persistent analgesic effects. Furthermore, EA can downregulate TRPV1-mediated inflammation and the release of proinflammatory cytokines, GFAP, Iba-1, and S100B by inhibiting the phosphorylation of p38 MAPK, therefore inhibiting microglial activation that triggers nerve damage and pain. Combining acupuncture to the treatment of a cerebral ischemic/hemorrhagic event can have a positive effect; an upregulation of BDNF synthesis, downregulation of P2X4Rs, and inhibition of p38 phosphorylation leads to the activation of the Raf-1/MEK1/2/ERK1/2/p90RSK/Bad signaling pathway, which contributes to the downregulation of caspase-3-dependent neuronal apoptosis. Acupuncture also inhibits the TNF-α/TRADD/FADD/cleaved caspase-8/cleaved caspase-3 apoptotic pathway in the ischemic cortical penumbra after reperfusion. In the case of treating epilepsy and seizure, several antiepileptic mechanisms have been proposed, including the downregulation of JNK or ERK1/2 and proinflammatory factors (IL-1β, IL-6, TNF-α) in the cerebral cortex and hippocampus. Auricular EA can reduce hippocampal hyperactivity through TRPA1 and PKCε/PKCα/pERK1/2 signaling pathways. Furthermore, acupuncture can regulate p38γ and p38δ and reduce Tau hyperphosphorylation in neurodegenerative diseases. Acupuncture also exhibits a therapeutic effect that was reported to involve the regulation of DJ-1, ASK1, Daxx, phosphorylated PKA, PKC, α-Ca2+/CaMKII, pERK, and CREB. Acupuncture exerts curative effects in migraine through local analgesia, anti-inflammation, neuropeptide regulation, cytoskeleton remodeling, cell repair, and improvement in overall homeostasis. Furthermore, the downregulation of TRPV4, p-p38, p-JNK, and pCREB in the peripheral nervous system and CNS may account for the therapeutic effect of acupuncture in fibromyalgia.

This literature review based on the results of previous studies where we electronically searched databases including PubMed, Embase, and the Cochrane Library from their inception to April 2020, using the following keywords alone or in various combinations: “acupuncture”, “p38 MAPK pathway”, “signaling”, “stress response”, “inflammation”, “immune”, “pain”, “analgesic”, “cerebral ischemic injury”, “epilepsy”, “Alzheimer’s disease”, “Parkinson’s disease”, “dementia”, “degenerative”, and “homeostasis”. The schematic explanation of the searching process is presented as Appendix A.

## 13. Conclusions

Among the aforementioned cell pathways, the p38 MAPK signaling pathway plays a role in the therapeutic effect of acupuncture in several nervous system diseases. EA inhibits the ascending pain pathway and intracellular p38-mediated inflammatory pathway by stimulating peripheral opioid receptors. Furthermore, EA promotes endogenic analgesic mechanisms, thus exerting immediate local analgesic effects and rescuing CNS-induced chronic pain. Acupuncture also counteracts p38 MAPK signal transduction, reduces Aβ deposits in the brain, and improves learning and memory in AD patients.

Acupuncture exhibits a dual regulatory function through the activation or inhibition of different p38 MAPK pathways and contributes to an overall improvement of clinical symptoms and physiological functions in several nervous system diseases. The p38 MAPK pathway potentially induces both protective and inhibitory effects in highly similar systems. Further studies are required to identify and characterize the various substrates of these kinases involved in cell differentiation/cell repair, neurotoxicity, and neurodegeneration, thus potentially furthering the current understanding of the mechanism underlying acupuncture in treating these diseases.

## Figures and Tables

**Table 1:** Members of the p38 mitogen-activated protein kinase (MAPK) subfamily.

p38 Subfamily	Other Names	Upstream	Location/Function	Dysfunction and Diseases
p38α	MAPK14, SAPK2a, CSBP	MKK3, MKK4, MKK6, MKK7	Ubiquitously expressed at significant levels in most cell types. Involved in the regulation of cell proliferation, differentiation, development, and response to stress [77,78,84,99].	Defective placental angiogenesis causing embryo death (mouse), symmetric synchronous cell cleavage (zebrafish), reduction in erythropoietin (Epo) production [99], leading to anemia, the impairment of glucogenesis (mice), and lipid-induced insulin-resistance (rat) [77,97].
p38β	MAPK 11	MKK3, MKK4, MKK6	Ubiquitously expressed; upregulated in the CNS and lungs, downregulated in the healthy heart [104,105,106].	No phenotype found [77].
p38γ	MAPK 13, ERK6, SAPK3 ^1^	MKK3, MKK4, MKK6MKK7	Myoblast and skeletal muscle./(1) Under stress conditions, they act on scaffold proteins targeting the plasma membrane cytoskeleton at sites of neuromuscular junctions and gap junctions [107]. (2) Recent studies indicate that the microbial metabolite imidazole propionate may contribute to the pathogenesis of type 2 diabetes via the activation of p38γ/p62/ mTORC1 [89].	No phenotype found (mouse) [77]. Meiotic G2/M progression of oocytes (xenopus) [77,104,105].
p38δ	MAPK 12, SAPK4	MKK3, MKK4, MKK6, MKK7	Only expressed in the lungs, kidney, testis, spleen, pancreas, and small intestine in humans, rats, and mice, but not in other vertebrates; enriched in endocrine glands [107,108]./(1) Regulates cytoplasmic microtubule dynamics, including tau protein [107]. (2) Upregulated in the liver in obese patients with NAFLD [89].	No phenotype found [77].

SAPK: stress-activated protein kinase; CNS: central nervous system; mTORC1: mammalian target of rapamycin complex 1 or mechanistic target of rapamycin complex 1; NAFLD: nonalcoholic fatty liver disease.

**Table 2:** The effect of acupuncture on inflammatory and neuropathic pain.

Study	Model	Intervention	Acupoints	Evaluation	Result
Hsu et al., (2014) [173]	SD rats	CCI-induced neuropathic pain; EA, 2- and 15-Hz, 20 min	Ipsilateral ST36-ST37 of the affected limb	Behavioral responses to stimuli; expression of TRPV1/4 in the cerebral cortex and lumbar spinal cord	EA relieved neuropathic pain; downregulation of cerebral TRPV4 expression.
Jiang et al., (2018) [174]	SD rats	CCI-induced neuropathic pain; EA, 2- and 15-Hz, 20 min	Bilateral L4-L6 Hua Tuo Jia Ji (EX-B2)	GABA_A_, A1R, TRPV1/4, and mGluR3 in the DRG	EA reduced the pain response, upregulating the GABA_A_ receptor in the spinal cord.
Huang et al., (2019) [175]	SD rats	CCI-induced neuropathic pain; EA, 2-, 15- and 50-Hz, 20 min	GV20, GV14	Expression of the GABA_A_ receptor and the level of glutamate in the hippocampus and periaqueductal gray (PAG) area.	EA reduced the pain response; suppressed hippocampal GABA_A_ receptors; decreased thalamic glutamate levels.
Lin et al., (2002) [176]	Human	Preoperative EA, 2- (low) or 100- (high) Hz, 20 min	Bilateral ST36	Postoperative pain and opioid-related side effects	Both low- and high-frequency EA reduced postoperative analgesic requirements and associated side effects.
Wang et al., (1997) [177]	Human	Postoperative TAES, 2- (low) or 100- (high) Hz, 30 min	Bilateral LI4	Postoperative pain and opioid-related side effects	Both low- and high-frequency EA reduced postoperative analgesic requirements and associated side effects.
Chen et al. (2011) [178]	CD1 mice	EA, 2-Hz, 20 min	Bilateral ST36	Behavioral responses in the paw and ASIC3 overexpression in DRG neurons.	Rescued mechanical hyperalgesia and an ASIC3 downregulation.
Chen et al. (2012) [179]	ICR mice	EA, 2-Hz, 15 min	Bilateral ST36	Behavioral responses in the paw and TRPV1/4 overexpression in DRG neurons.	TRPV1 and TRPV4 upregulation in DRG neurons was attenuated by EA.
Huang et al. (2013) [180]	ICR mice	EA, 2-Hz, 15 min	Bilateral ST36	Behavioral responses in the paw and the overexpression of Na_v_1 in DRG neurons.	EA attenuated inflammatory pain by suppressing Na_v_1 overexpression.
Wu et al. (2014) [181]	ICR mice	MA, 60 min	Ipsilateral ST36 of the inflamed limb	Behavioral responses in paw; the overexpression of TRPV1/4, ASIC3, and CWP components in the anatomical layers of ST36.	MA induced analgesia, with high TRPV1 and CWP overexpression at ST36 upon MA.
Lu et al. (2016) [182]	C57/B6 mice	EA, 2-Hz, 15 min	Ipsilateral and contralateral ST36-ST37 of the inflamed limb	Behavioral responses in the paw; Na_v_ and TRPV1 overexpression in DRG neurons.	Hyperalgesia was suppressed through ipsilateral and contralateral EA. Na_v_ and TRPV1 were suppressed through EA.
Liao et al. (2017) [143]	C57/B6 mice	EA, 2-Hz, 15 min	Bilateral ST36	Behavioral responses in the paw and the expression of Na_v_, GFAP, Iba-1, S100B, RAGE, and TRPV1 in DRG neurons.	EA attenuated inflammatory pain by suppressing Nav1.8 through S100B, TRPV1, opioid, and adenosine pathways.
Liao et al. (2017) [183]	C57/B6 mice	EA, 2-Hz, 15 min	Bilateral ST36	Behavioral responses in the paw and the expression of GFAP, S100B, RAGE, PKCε, ERK, NF-κB, and COX-2 in DRG neurons.	EA attenuated inflammatory pain by suppressing opioid and adenosine pathways.
Yang et al. (2017) [184]	C57/B6 mice	EA, 2-Hz, 15 min	Bilateral ST36	Behavioral responses in the paw and the expression of TRPV1, PKA, PKC, PI3K, ERK1/2, p38, JNK, Akt, mTOR, CREB, NF-κB, Na_v_1.7/1.8, GFAP, S100B, and RAGE in DRG neurons.	EA significantly reduced chronic inflammatory pain by downregulating the TRPV1 pathway from the peripheral DRG neurons to the central spinal cord.
Yen et al. (2019) [185]	C57/B6 mice	EA, 2-Hz, 15 min	Bilateral LI4	Behavioral responses in the paw and the expression of TRPV1 and ERK1/2 in the prefrontal cortex, the hypothalamus, the PAG area, and DRG neurons.	Pain alleviation immediately after EA; the expression of TRPV1-associated molecules was attenuated by EA in the prefrontal cortex, the hypothalamus, the PAG area, and DRG.
Hsu et al. (2019) [186]	C57/B6 mice	EA, 2-Hz, 15 min	Bilateral ST36	Behavioral responses in the paw and the expression of TLR2, PI3K, ERK1/2, p38, JNK, Akt, mTOR, CREB, NF-κB, and Na_v_1.7/1.8 in the thalamus.	EA attenuated inflammatory pain via TLR2 signaling.
Yang et al. (2009) [187]	Patients with CTS	MA, 30 min/session, 2 session a week, 8 session in total	Affected side(s), PC6, PC7	Motor and sensory NCS; designed symptomatic questionnaire.	Short-term acupuncture was as effective as short-term low-dose steroid for mild-to-moderate CTS.
Yang et al. (2011) [188]	Patients with CTS	MA, 30 min/session, 2 session a week, 8 session in total	Affected side(s), PC6, PC7	NCS; global symptom score.	Acupuncture had superior efficacy to steroid treatment not only in terms of objective changes in nerve conduction but also in terms of subjective symptom assessment in long-term follow-up.
Yang et al. (2011) [189]	Patients with chronic migraine (CM)	MA, 30 min/session, 2 session a week, 24 session in total	Bilateral BL2, GB20, EX-HN5, EX-HN3 (acupoints relate to the trigeminal and cervical dermatomes)	Changes in headache events, MIDAS scores, HADS scores, BDI-II scores, reduction of medication.	Acupuncture was similarly effective or more effective than prophylactic drug treatment with less side effects in migraine.

A1R: adenosine A1 receptor; ASIC3: acid-sensing ion channel; BDI-II: Beck Depression Inventory-II; CCI: chronic constriction injury; CWP components: components of calcium wave propagation, including pannexin 1, connexin 43, P2Y1, and P2Y2, which can activate a release of ATP after mechanical stimulation of nonneural cells such as subepithelial fibroblasts; GABA_A_: γ-aminobutyric acid A; GFAP: glial fibrillary acidic protein, an astrocytic marker; HADS: hospital anxiety and depression scale; Iba-1: ionized calcium-binding adaptor molecule 1, a microglia/macrophage specific protein (marker); MA: manual acupuncture; MIDAS: Migraine Disability Assessment; mGluR3: metabotropic glutamate receptor 3; Na_v_s: voltage-gated sodium channels; NCS: nerve conduction study; RAGE: receptor for advanced glycation end-products; TAES: transcutaneous acupoint electrical stimulation; 100B: calcium-binding protein B.

**Table 3:** The p38 signaling pathway in cerebral ischemic stroke.

Study	Model	Intervention	Acupoints	Evaluation	Result
Bäcker et al., (2003) [13]	Healthy human	MA; manipulation as either high frequency (4–8 Hz) and low amplitude (Hf–La) or low frequency (1–2 Hz) and high amplitude (Lf–Ha).	Right LI4	Cerebral blood flow velocity (CBFV) in both middle cerebral arteries, arterial blood pressure (BP), heart rate (HR).	(1) Lf–Ha stimulation was perceived as more intense and markedly increased the CBFV in the right hemisphere; (2) Hf–La stimulation slightly decreased BP and HR; (3) Lf–Ha stimulation induced an initial pressor response (increase of BP, decrease of HR) and a more marked long-term BP reduction.
Hsieh et al., (2006) [14]	SD rats	EA, 2-Hz, 15 min	Both ST36	The levels of nitric oxide in the peripheral blood and amounts of calcitonin gene-related peptide (CGRP) in the cerebral cortex and thalamus. L-N (G)-nitro arginine methyl ester (L-NAME) was used to measure the changes in CBF.	Both 2- and 15-Hz EA increased CBF in rats with and without CI.
Cheng et al. (2014) [147]	SD rats	EA, 2-Hz, 25 min once daily for 2 consecutive days.	GV20, GV14	Cerebral infarct area, caspase-3, BDNF, pRaf-1, MEK1/2, ERK1/2, p90RSK, and Bad.	EA significantly reduced the cerebral infarct area, caspase-3 protein expression levels, and apoptosis in the ischemic cortex. BDNF, phospho-Raf-1 (pRaf-1), phospho-MEK1/2 (pMEK1/2), phospho-ERK1/2 (pERK1/2), phospho-90 kDa ribosomal S6 kinase (pp90RSK), and phospho-Bad (pBad) were markedly upregulated, and neuronal nuclear antigen (NeuN) expression was restored.
Cheng et al. (2014) [161]	SD rats	EA, 2-Hz, 15 min once daily for 6 consecutive days.	GV20, GV14	Cerebral infarct area, GFAP, S100B, NF-κB, p50, p38 MAPK, TNF-α, and iNOS.	EA significantly reduced the cerebral infarct area and downregulated astrocytic S100B expression and decreased p-p38 NF-kB.
Cheng et al. (2015) [203]	SD rats	EA, 5- or 25-Hz, 30 min once daily for 7 consecutive days.	GV20, GV16	Cerebral infarct area, GFAP, Bax, Bcl-xL, Smac/DIABLO, p-p38, and CREB.	Both 5- and 25-Hz EA effectively downregulated reactive astrocytosis to exert neuroprotective effects against cerebral infarction, most likely by activating the p38 MAPK/CREB signaling pathway.
Xu et al. (2014) [208]	SD rats	EA, 2-Hz, 20 min once a day.	GV20, ST36	Hsp70 and TNF-α peripheral serum.	Lowered peak levels of adrenocorticotrophic hormone and Hsp70.
Kuo et al. (2016) [15]	SD rats	Electrostimulation, 2-Hz, 20 min once daily for 7 consecutive days.	Both ears	Brain nicotinic acetylcholine receptors.	Two-hertz ES for ameliorated learning and memory impairment.

GFAP: glial fibrillary acidic protein; iNOS: inducible nitric oxide synthase; MA: manual acupuncture; Smac/DIABLO: second mitochondrial-derived activator of caspase/direct inhibitor of apoptosis protein-binding protein with low isoelectric point.

**Table 4:** The effect of acupuncture on epileptic seizures.

Study	Model	Intervention	Acupoints	Evaluation	Result
Kim et al., (2008) [215]	ICR mice	MA, 20 min/day, for 2 days	Bilateral HT8	Hippocampal expression of c-Fos, c-Jun, and GAD-67 (CA1 and CA3 areas).	Reduced severity of epileptic seizures and the rate of neuronal death; downregulation of c-Fos and c-Jun; upregulation of GAD-67.
Kim et al. (2012) [216]	C57BL/6 mice	MA, 20 min/day, for 2 days	Bilateral HT8	Neuronal survival, microglial and astrocyte activation, and hippocampal mRNA expression of IL-1β and TNF-α.	Inhibition of hippocampal cell death and suppression of KA-induced inflammatory events.
Bae et al. (2013) [217]	C57BL/6 mice	MA, 20 min/day, for 3 days	Bilateral HT8	Neuronal survival and hippocampal astrocyte activation.	Acupuncture altered hippocampal protein expression to promote neuronal survival.
Liu et al. (2014) [218]	SD rats	EA, 2 Hz, 30 min/day for 7 consecutive days.	Bilateral ST-36-ST37 and ears	Changes in mossy fibers sprouting in the hippocampus.	Amelioration of mossy fibers sprouting in the hippocampus.
Lin et al., (2014) [220]	SD rats	EA, 2 Hz, 20 min/day, 3 days/week for 6 weeks	Bilateral ears, ST36, ST37	EEG and EMG changes; hippocampal TRPA1, TRPV4, PKCα, PKCε, and pERK1/2 expression.	EA reduced hippocampal hyperactivity accompanied by alterations in the TRPA1, PKCε, PKCα, and pERK1/2 signaling pathways.
Liao et al., (2017) [221]	SD rats	EA, 2 Hz, 20 min/day, 3 days/week for 6 weeks	Bilateral ears, ST36, ST37	EEG and EMG changes; hippocampal COX-2 levels.	Attenuated COX-2 and COX-2 immunoreactive cells in the hippocampal CA1 region after epileptic seizures.
Liao et al. (2018) [40]	SD rats	EA, 2 Hz, 20 min/day, 3 days/week for 6 weeks	Bilateral ears	Brain TLR4, CaMKIIα, ERK, JNK, and NF-κB expression.	Auricular EA controlled epileptic seizures by regulating the TLR4 signaling pathway.

GAD-67: glutamate decarboxylase-67; c-Fos, c-Jun: proto-oncogenes that are expressed within some neurons following depolarization, the two form the AP-1 early response transcription factor that regulates gene expression in response to extracellular stimuli; KA: kainic acid; EEG: electroencephalogram; EMG: electromyogram; PKC: protein kinase C; TRPA1: transient receptor potential cation channel subfamily A member 1; TRPV: transient receptor potential vanilloid receptors; pERK: phosphor-extracellular signal-regulated kinases; COX-2: cyclooxygenase-2; TLR4: Toll-like receptor 4; CaMKIIα: calmodulin-dependent protein kinase II alpha.

**Figure A1:**
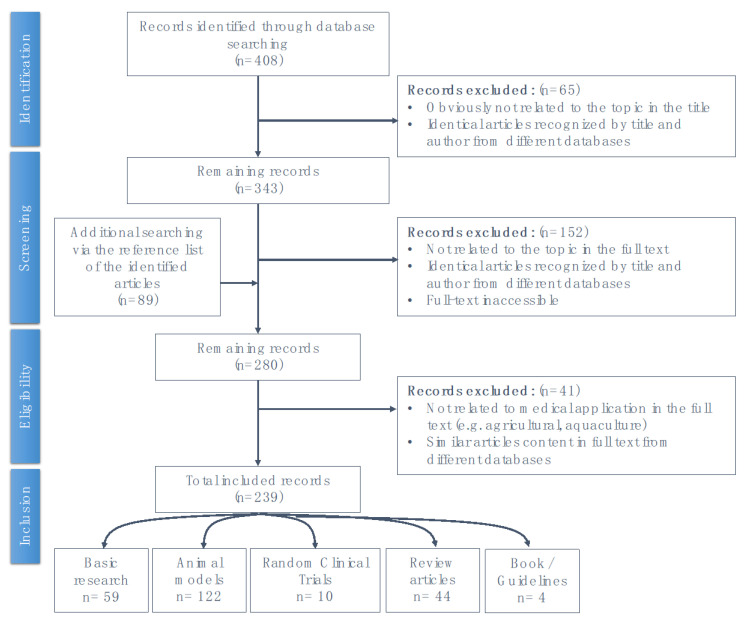
Schematic of the literature review search process.

## Abbreviations

A1R	adenosine A1 receptor
α7 nAChR	α7 nicotinic acetylcholine receptor
AC	adenyl cyclase
ACC1	acetyl-CoA carboxylase (ACC) 1 is a biotin-dependent enzyme that catalyzes the irreversible carboxylation of acetyl-CoA to produce malonyl-CoA
AD	Alzheimer’s disease
Akt (PKB)	protein kinase B (PKB) is also known as Akt
Alk5	TGFβ type I receptor kinase
AMP	adenosine mono-phosphate
AMPK	5’ AMP-activated protein kinase
AP-1	activator protein 1
ARE	AU-rich elements
ASIC3	acid-sensing ion channel 3
ASK-1	apoptosis signal-regulating kinase 1
ATF	activating transcription factor
ATP	adenosine tri-phosphate
BACE1	beta-secretase 1, an enzyme responsible for Aβ generation in Alzheimer’s disease
Bad	BCL2 associated agonist of cell death; a protein involved in initiating apoptosis.
Bax	Bcl-2 associated X
Bac-2	B-cell lymphoma 2
BDNF	brain-derived neurotrophic factor
CaMKII	calmodulin-dependent protein kinase II
CCI	chronic constriction injury
Cd242	The 40- to 42-kiloDalton red cell membrane glycoprotein bearing the ICAM-4 antigen named by the LW blood system.
CFA-treated	Complete Freund’s adjuvant injections produced significant mechanical and thermal hyperalgesia in mice.
c-Fos	A proto-oncogene that is expressed within some neurons following depolarization.
CHOP	C/EBP homologous protein; belongs to the family of CCAAT/enhancer-binding proteins (C/EBPs) and is involved in the regulation of genes that encode proteins involved in proliferation
c-Jun	A proto-oncogene that with c-Fos forms the AP-1 early response transcription factor that regulates gene expression in response to extracellular stimuli.
CL100	The human CL100 gene is induced in skin fibroblasts in response to oxidative/heat stress and growth factors. The CL100 gene encodes a dual specificity (Tyr/Thr) protein phosphatase that specifically inactivates MAPKs.
CLCA3A2	chloride channel accessory 3A2
CLIC3	chloride intracellular channel 3
Cot (Tp2)	cancer Osaka thyroid oncogene (=Tpl-2)
COX-2	cyclooxygenase-2
cPLA2	cytosolic phospholipase A2
CREB	cAMP response element-binding protein
CRF	corticotropin-releasing factor
CWP	components of calcium wave propagation
DLK (MAP3K12)	dual leucine zipper kinase, also known as MAP3K12
DJ-1	a protein deglycase
DLG1 (SAP97)	discs large homolog 1 scaffold protein
Daxx	the death-associated protein
DHT	dihydrotestosterone
DRG	dorsal root ganglion
EA	electroacupuncture
elF2a	eukaryotic translation initiation factor 2A; functions by a separate mechanism in eukaryotic translation
eEF2	eukaryotic elongation factor 2
eEF2K	eukaryotic elongation factor 2 kinase
elF2α	a subunit of the heterotrimeric eIF2 complex
Elk-1	erythroblast transformation specific (ETS) like-1 protein
ERK	extracellular signal–regulated kinases
ETS	erythroblast transformation specific protein, or E26 transformation-specific, or E-twenty-six transcription factors family
FADD	Fas-associated protein with death domain; also called MORT1
Fas pathway	Fas and Fas Ligand (FasL) are involved in the regulation of cell death.
GABA	gamma-aminobutyric acid
GFAP	glial fibrillary acidic protein; a marker of astrocytes
GPCRs	G-protein-coupled receptors
HMG-CoA Reductase	3-hydroxy-3-methyl-glutaryl-CoA reductase or HMGR is the rate-controlling enzyme of the mevalonate pathway, responsible for cholesterol and other isoprenoid biosynthesis.
Hsc70	heat shock cognate 70
Hsp	heat shock proteins
5-HT	5-hydroxytryptamine
Iba-1	ionized calcium–binding adapter molecule 1
IκB	inhibitor of nuclear factor kappa-B
IκB	inhibitor of nuclear factor kappa-B kinase
IKKs	I-kappa-B kinases
IL-1β	Interleukin 1 beta is a member of the interleukin 1 family of cytokines produced by activated macrophages.
IRF	interferon regulatory factor
JNK	c-Jun amino-terminal kinases
LTD	long-term depression; a term in neurophysiology describing an activity-dependent reduction in the efficacy of neuronal synapses lasting hours or longer following a long patterned stimulus.
LTP	long-term potentiation; a persistent strengthening of synapses based on recent patterns of activity. These are patterns of synaptic activity that produce a long-lasting increase in signal transmission between two neurons.
MA	manual acupuncture
MAPK	mitogen-activated protein kinase
MAPKK (MAP2K, MKK)	MAPK kinase
MAPKKK (MAP3K)	MAPKK kinase
Max	a transcription factor coded by the myc-associated factor X
MEF	myocyte enhancing factor
MEK (MKK)	MAPK/ERK kinase
MEKK	MEK kinase
mGluR3	metabotropic glutamate receptor 3
MKP	MAPK-phosphatase
MAPKAPK-2	mitogen-activated protein kinase-activated protein kinase 2
MLK	mixed-lineage protein kinase
MNK	mitogen activated protein kinase–interacting protein
Myc	A group of transcription factors coded by a regulator genes and proto-oncogenes called Myc
MyD88	myeloid differentiation primary response 88
MSK	mitogen and stress activated protein kinase
mTOR	mammalian target of rapamycin
Na_v_	voltage-gated sodium channel
NFκB	nuclear factor-kappa-B
P105	The 105 kD protein is a Rel protein-specific transcription inhibitor encoded by the NFKB1 gene
PAG	periaqueductal gray area
NIH3Ts cells	A cell line derived from mouse embryonic fibroblasts.
NMDA	N-methyl-D-aspartate, an amino acid derivative
PKB	Protein kinase B; also known as Akt
NO	nitric oxide
NR1	NMDA receptor subunits includes NR1 and NR2Bi
Pax6	Paired-box protein Pax-6, also known as aniridia type II protein (AN2) or oculorhombin; a “master control” gene for the development of eyes and other sensory organs
P2RX7	P2X purinoceptor 7 is a protein belonging to the family of purinoceptors for ATP.
P2X4Rs	P2X4 receptors are a subtype of ionotropic ATP receptors
P2Y1, -2	human purinergic G protein-coupled receptors
PD	Parkinson’s disease
Pellino	A group of proteins extremely well conserved during evolution; Pellinos interact with key mediators in TLR/IL-1R-induced signaling pathways.
PHF-tau	paired helical filament–tau protein
PI3K	phosphoinositide 3-kinases; also called phosphatidylinositol 3-kinases
PKC	protein kinase C
PSD	postsynaptic density protein
PRAK	p38-regulated and -activated kinase
Rac2	Ras-related C3 botulinum toxin substrate 2
RAF	rapidly accelerated fibrosarcoma-related oncogene
Raf1	v-raf-1 murine leukemia viral oncogene homolog 1
RAGE	The receptor for advanced glycation end products is a member of the immunoglobulin superfamily of cell surface molecules.
RCT study	randomized controlled trial study
Rel	A proto-oncogene protein encoded by the REL gene. It is a member of the NF-κB family of transcription factors and contains a Rel homology domain (RHD) at its N-terminus and two C-terminal transactivation domains.
Rho	Rho (ρ) factor is a protein that acts in bacterial cells to mediate termination of transcription at distinct sites, which mediates the dissociation of the RNA from the very stable ternary transcription complex.
Rit	Ras-like protein in tissues
RIP	receptor-interacting protein kinases; a class of serine/threonine protein kinases
ROS	reactive oxidative species
S100B	S100 calcium-binding protein B
SAP	stress-activated protein or synapse-associated protein; SAP90 [(synapse-associated protein 90 is also known as PSD-95 (postsynaptic density-95)]
SAPK	stress-activated protein kinase
Smac/DIABLO	second mitochondrial-derived activator of caspase/direct inhibitor of apoptosis protein-binding protein with low isoelectric point
SNpc	substantia nigra pars compacta
Stat1	signal transducer and activator of transcription 1
STK	serine-threonine/tyrosine kinases
TAES	transcutaneous acupoint electrical stimulation
TAK-1	transforming growth factor-β-activated kinase 1
TAO	thousand and one amino acids
TAB1	TGF-beta-activated kinase
TGFβ	transforming growth factor-β
TLR4	toll-like receptor 4
TNF	tumor necrosis factor
Tpl2	tumor progression locus 2, also known as COT or MAP3K8
TRADD	tumor necrosis factor receptor type 1-associated DEATH domain protein
TRAF6	tumor necrosis factor receptor (TNFR)-associated factor 6
TrkB	tyrosine kinase receptor B
TRPA1	transient receptor potential cation channel subfamily A member 1
TRPV	transient receptor potential vanilloid receptors
3′UTR	3′untranslated region
